# Enhancement Methods of Antioxidant Capacity in Rice Bran: A Review

**DOI:** 10.3390/foods11192994

**Published:** 2022-09-26

**Authors:** Riza Andriani, Toto Subroto, Safri Ishmayana, Dikdik Kurnia

**Affiliations:** Department of Chemistry, Faculty of Mathematics and Natural Science, Universitas Padjadjaran, Sumedang 45363, Indonesia

**Keywords:** rice bran, *japonica*, *indica*, by-products, phenolic compound, bioactive compound

## Abstract

Rice (*Oryza sativa* L.) is a primary food that is widely consumed throughout the world, especially in Asian countries. The two main subspecies of rice are *japonica* and *indica* which are different in physical characteristics. In general, both *indica* and *japonica* rice consist of three types of grain colors, namely white, red, and black. Furthermore, rice and rice by-products contain secondary metabolites such as phenolic compounds, flavonoids, and tocopherols that have bioactivities such as antioxidants, antimicrobial, cancer chemopreventive, antidiabetic, and hypolipidemic agents. The existence of health benefits in rice bran, especially as antioxidants, gives rice bran the opportunity to be used as a functional food. Most of the bioactive compounds in plants are found in bound form with cell wall components such as cellulose and lignin. The process of releasing bonds between bioactive components and cell wall components in rice bran can increase the antioxidant capacity. Fermentation and treatment with enzymes were able to increase the total phenolic content, total flavonoids, tocotrienols, tocopherols, and γ-oryzanol in rice bran.

## 1. Introduction

Chronic diseases such as cancer, diabetes mellitus, stroke, and cardiovascular disease are characterized by an increase in oxidative stress status [1,2,3,4]. Oxidative stress is caused by an imbalance between the production and neutralization of free radicals in biological systems [5]. Free radicals usually come from reactive oxygen species (ROS), reactive nitrogen species (RNS), and reactive sulfur species (RSS) [6].

The free radicals produced can be neutralized in the presence of antioxidants, so it can prevent oxidative damage in the human body [5]. Many studies have reported that rice is rich in antioxidants [7,8,9,10,11,12]. In addition, rice bran is a by-product obtained after milling and also is rich in antioxidants, but previously it was used only as animal feed [13,14,15,16].

Rice (*Oryza sativa* L.) is widely cultivated in Asia and is important in the world because it is used as a primary food. This species consists of two main subspecies, namely *japonica* and *indica*; the difference between the two lies in morphological and agronomic characteristics, in physiological and biochemical characteristics, and in genomic structure [17]. In general, rice consists of three types of grain colors, namely white, red, and black. However, in some areas such as Thailand, there are also types of brown and purple rice [18,19,20].

In white, red, and black rice, secondary metabolites such as phenolic acids and anthocyanins are highest in rice bran compared with other parts of rice such as whole grains, embryos, and endosperm [21]. Several studies reported that secondary metabolites in rice bran have bioactivity as antioxidants, antimicrobial, cancer chemopreventive, antidiabetic, and hypolipidemic agents [22,23,24,25]. However, secondary metabolites such as phenolics exist in bound form which is insoluble and make it difficult to extract [26]. Therefore, several current studies are focusing on the release of bound phenolics so that their bioactivity is increased [27,28]. Previous research reported that rice bran can be used as functional foods such as oil, milk, flour, and bread [29,30,31].

Prior to the isolation of natural products, an extraction process is carried out to separate the bioactive components from the raw material. The extraction technique consists of solvent and non-solvent extraction. The solvents used are methanol, ethanol, acetone, chloroform, hexane, ethyl acetate, dichloromethane, and isopropanol. The solvent extraction method is more widely used but the solvent used is toxic. Therefore, there are alternative extraction methods such as ultrasound extraction (UAE) and supercritical fluid extraction (SFE) [32]. The most widely used isolation method is column chromatography using silica [33]. Isolated bioactive substances are generally identified and characterized using HPLC (High Performance Liquid Chromatography) [26], TLC (Thin Layer Chromatography [34], FTIR (Fourier-transform infrared spectroscopy), NMR (Nuclear magnetic resonance), etc. [35]

On the basis of this, this review focused on the antioxidant activity found in rice bran of rice (white, red, and black) varieties that have been extensively studied. In contrast with white rice bran, pigmented rice bran such as red and black rice contains anthocyanins which have antioxidant activity [21]. The antioxidant activity of bioactive compounds is caused by the presence of a hydroxyl group on the phenol group which is able to donate hydrogen atoms to free radicals [36]. This review will also discuss several methods that were used to increase the antioxidant capacity of rice bran.

## 2. Comparison of *Japonica* and *Indica*

*Japonica* is widely consumed by the people of China, Japan, South Korea, the United States, and Egypt [37]. The grain cultivar is long with a bold shape. In general, the amylose and total carbohydrate content are lower than the *indica*, but the protein, lipid, and dietary fiber content are higher than the *indica. Japonica* have length, width, and elliptic factor of 7.40; 3.71; and 1.99 mm, respectively. [38]. Ding et al. [39] conducted a study comparing *japonica* and *indica* rice grown in the same area in China, showing that *japonica* rice bran and endosperm is higher in total phenolic content than *indica* rice. The antioxidant activity also shows higher yields in *japonica* rice.

**Figure 1:**
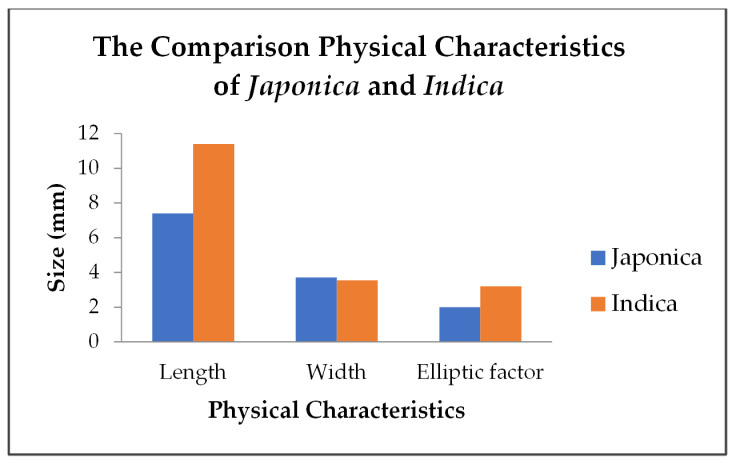
The comparison physical characteristics of *japonica* and *indica*.

*Indica* is widely consumed in Southeast Asian countries such as Vietnam, Thailand, Indonesia, Malaysia, Myanmar, and the Philippines [37]. The grain cultivar is extra long with a slender shape. The grains of this variety have a higher area (mm^2^), circumference (mm), and length (mm) than *japonica* shown in Figure 1 [38]. *Indica* have length, width, and elliptic factor of 11.34; 3.54; and 3.20 mm, respectively. In general, the amylose and total carbohydrate content are higher than in *japonica*, but the protein, lipid, and dietary fiber content are lower than in *japonica* [38].

## 3. Isolation Methods and Bioactive Compound Content in Rice Bran from Three Types of Grain Colors

### 3.1. White Rice Bran

Several methods have been carried out to isolate bioactive compounds in white rice bran. Peanparkdee et al. [40] used white rice bran with KDML 105 variety (indica rice) from Thailand. A total of 5 g of bran sample was dissolved in 100 mL of 65% (*v/v*) ethanol, isopropanol, and a mixture of isopropanol and hexane (1:1 *v/v*). The extracts of the three solvents were detected for the content of phenolic acids, flavonoids, and anthocyanins using high-performance liquid chromatography.

Aziz et al. [34] carried out extraction by reflux extraction using several solvents such as hexane, chloroform, ethyl acetate, dichloromethane, isopropanol, acetone, and a mixture of hexane-ethyl acetate (1:1), hexane isopropanol (1:1), and chloroform-ethyl acetate (1:1). In that study they used white rice bran from Banten Province, Indonesia. The TLC (Thin Layer Chromatography) densitometric method was used to determine the total oryzanol content. Then, column chromatography of the hexane extract was performed using silica gel 60 and gradient elution with hexane-ethyl acetate. As a result, six fractions were obtained, then the 5th fraction (F5) was subjected to centrifugal thin layer chromatography (CTLC) using silica gel GF_254_ and gradient elution using hexane-ethyl acetate to obtain 9 subfractions. Purification was carried out by centrifugal thin layer chromatography (CTLC) with isocratic elution using hexane-ethyl acetate (9:1) [34].

Pokkanta et al. [41] also carried out a study on KDML 105 white rice bran with another method to extract rice bran using 1:10 (*w/v*) methanol. Then, the mixture was centrifuged and the supernatant was taken. The methanol extract was extracted again in the same way using dichloromethane and hexane as solvents. The HPLC method was used to determine the content of tocols, γ-oryzanols, phytosterols, squalene, phylloquinone, and cholecalciferol [41]. Hansakul et al. [42] also used 80% methanol to extract rice bran. The type of white rice used was the 9311 variety [21]. In addition, there was also the extraction of rice bran by heating at 60–70 °C in distilled water 1:4 (*w/v*). Then, the extract was analyzed for total phenolic content by the Folin–Ciocalteu method and total flavonoid content [42].

**Figure 2:**
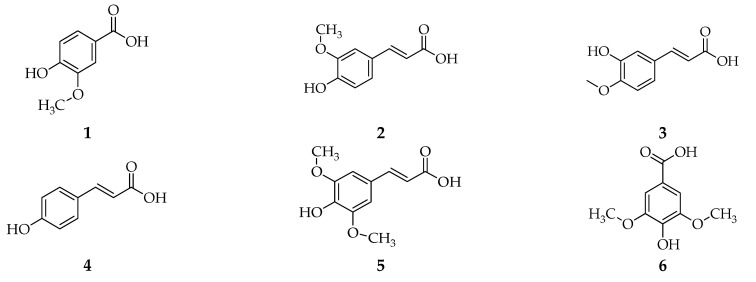

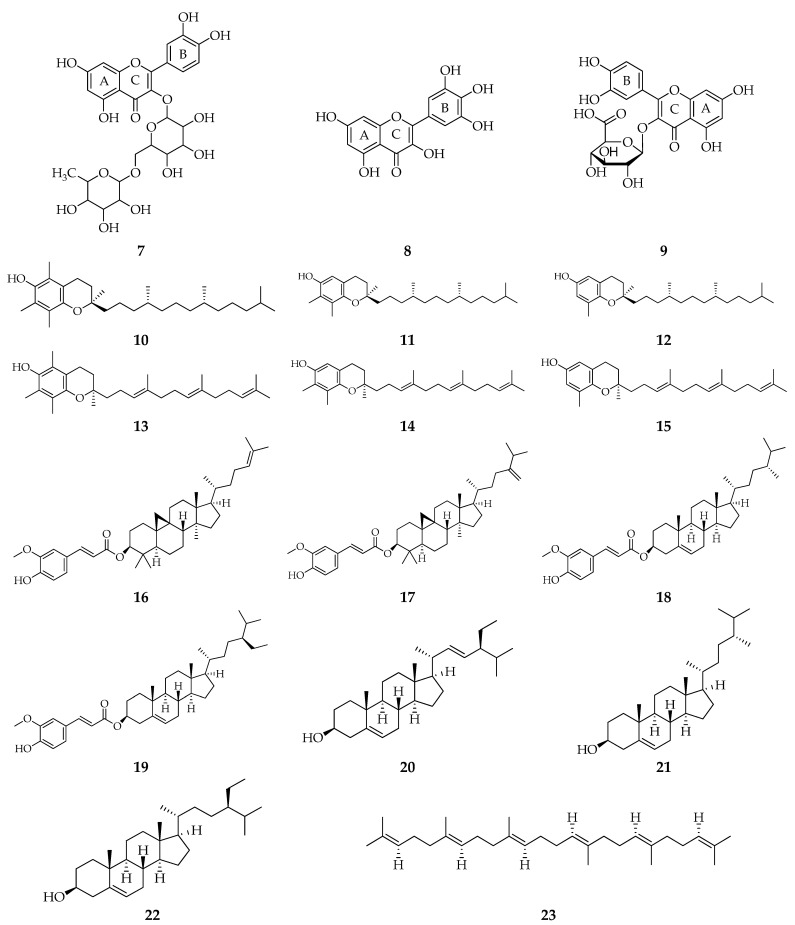
The compounds in different varieties of white rice bran use different isolation methods. (**1**) vanillic acid; (**2**) ferulic acid; (**3**) isoferulic acid; (**4**) p-coumaric acid; (**5**) sinapic acid; (**6**) syringic acid; (**7**) rutin; (**8**) myricetin; (**9**) quercetin-3-glucuronide [21,40]; (**10**) α-tocopherols; (**11**) γ-tocopherols; (**12**) δ-tocopherols; (**13**) α-tocotrienols; (**14**) γ-tocotrienols; (**15**) δ-tocotrienols; (**16**) cycloartenyl ferulate; (**17**) 24-methylene cycloartanyl ferulate; (**18**) campesteryl; (**19**) β-sitosteryl ferulate; (**20**) stigmasterol; (**21**) campesterol; (**22**) β-sitosterol; and (**23**) squalene [34,41].

From these methods, it is known that various varieties of white rice bran contain bioactive compounds such as phenolic (vanillic acid, ferulic acid, isoferulic acid, p-coumaric acid, sinapic acid, and syringic acid), flavonoid (rutin, myricetin, and quercetin-3-glucuronide) [21,40], tocols (α-tocopherols, α-tocotrienols, γ-tocopherols, γ-tocotrienols, δ-tocopherols, and δ-tocotrienols), γ-oryzanols (cycloartenyl ferulate, 24-methylene cycloartanyl ferulate, campesteryl ferulate, and β-sitosteryl ferulate), phytosterols (stigmasterol, campesterol, and β-sitosterol), and squalene [34,41] (Figure 2).

### 3.2. Red Rice Bran

Red rice also has many types of varieties that are reported to have bioactive components. Jakwangdobyeo red rice bran from Gusan, Korea, has an antioxidant component. Rice bran was extracted twice using acetone 1:20 (*w/v*), methanol 1:20 (*w/v*), and ethanol 1:20 (*w/v*) with a solvent concentration range of 0 to 100%. Then, it was centrifuged at 1000 × g for 15 min and filtered. The supernatant was dissolved to 400 mL with each extract solvent. Then, the DPPH (2,2-diphenyl-1-picrylhydrazyl) test was carried out on the extract, total phenolic content was determined using the Folin–Ciocalteu method, and total flavonoid content was determined based on the Jia et al. (1999) method. The results showed the highest antioxidant activity, total phenolic content, and total flavonoid content in 40% acetone extract. This 40% acetone extract was then analyzed for phenolic acid content using HPLC [43].

Moreover, Huang and Lai [44] used three samples of red rice bran. Two of the red rice bran samples were from Taibalang and Guangfu red rice varieties were obtained from Hualien, Taiwan. Meanwhile, another sample of rice bran was obtained by importing from Thailand, of which the varieties were not mentioned. All samples of red rice bran had free phenolic and bound phenolic extracted; extraction was carried out twice using 80% ethanol. The supernatant obtained was extracted with ethyl acetate to obtain free phenolic extract while the residue was dissolved in 80% ethanol and re-extracted with ethyl acetate to obtain the bound phenolic extract. Both extracts were analyzed for phenolic acid content using HPLC. This study also analyzed the content of proanthocyanin and anthocyanin using HPLC/ESI-MS/MS. Extraction was carried out using methanol. In addition, an analysis of the content of vitamin E and γ-oryzanol was carried out using normal phase HPLC. Extraction was carried out using hexane solvent containing 0.02% butylated hydroxytoluene [44].

Pengkumsri et al. [45] also analyzed Mali red rice bran obtained from Thailand. Rice bran was extracted using 80% ethanol. Phenolic compounds were determined using reversed-phase HPLC with gradient elution. Meanwhile, to determine the anthocyanin content using 0.1 N HCl in methanol as solvent, anthocyanin content was determined by reversed-phase HPLC [45]. Shao et al. [21] also used 80% methanol to extract rice bran. The type of red rice used was the SB7 variety.

Ghasemzadeh et al. [46] carried out a study on red rice bran obtained from the Rice Research Institute of Iran (RRII). The red rice bran had previously been defatted by heating and extraction using hexane. Digital electric heating thermostat circulating water bath with double line six holes was used for extraction. The solvents used were ethanol and acetone. This research optimized various extraction conditions such as extraction time, extraction temperature, solvent-to-solid ratios (S/S), and solvent percentage to produce the highest flavonoids. Then, the content of flavonoids and phenolic acids was identified using the Agilent HPLC 1200 system coupled to an API 3200 triple quadrupole mass spectrometer.

In addition to dry bran samples, determination of the content of bioactive compounds was carried out on bran oil products. Rice bran oil was made from Mali red rice bran and was extracted using hexane (1:10). This study used different extraction methods including hexane extraction, hot press extraction, cold press extraction, and supercritical fluid extraction. The best yield of rice bran oil extraction was shown by the hexane extraction method. The content of γ-oryzanol and tocols was determined by reversed-phase HPLC [47].

Moko et al. [35] isolated stigmasterol in red rice bran obtained from Minahasa Regency, North Sulawesi, Indonesia. Samples were extracted using ethanol and fractionated using solvents (hexane, ethyl acetate, and n-butanol). Then, Thin Layer Chromatography (TLC) was performed on the hexane extract using the stationary phase of silica gel 60 Merck and eluent hexane-ethyl acetate (9:1) with a 5% stepwise elution. Stigmasterol was identified using FTIR and NMR.

**Figure 3:**
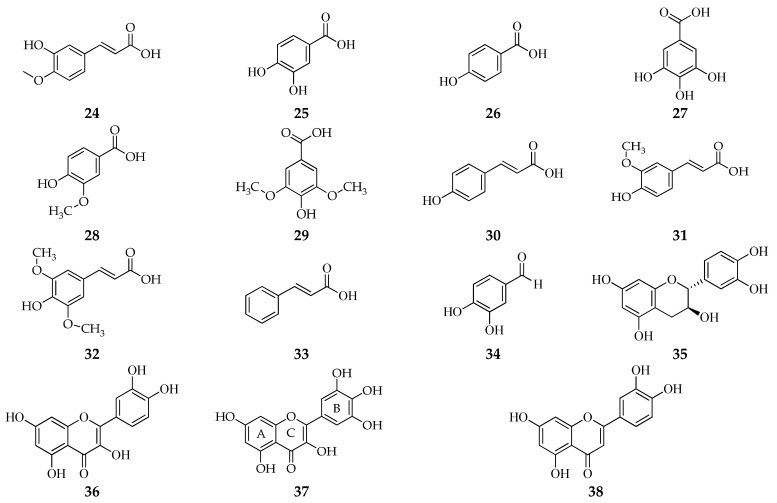

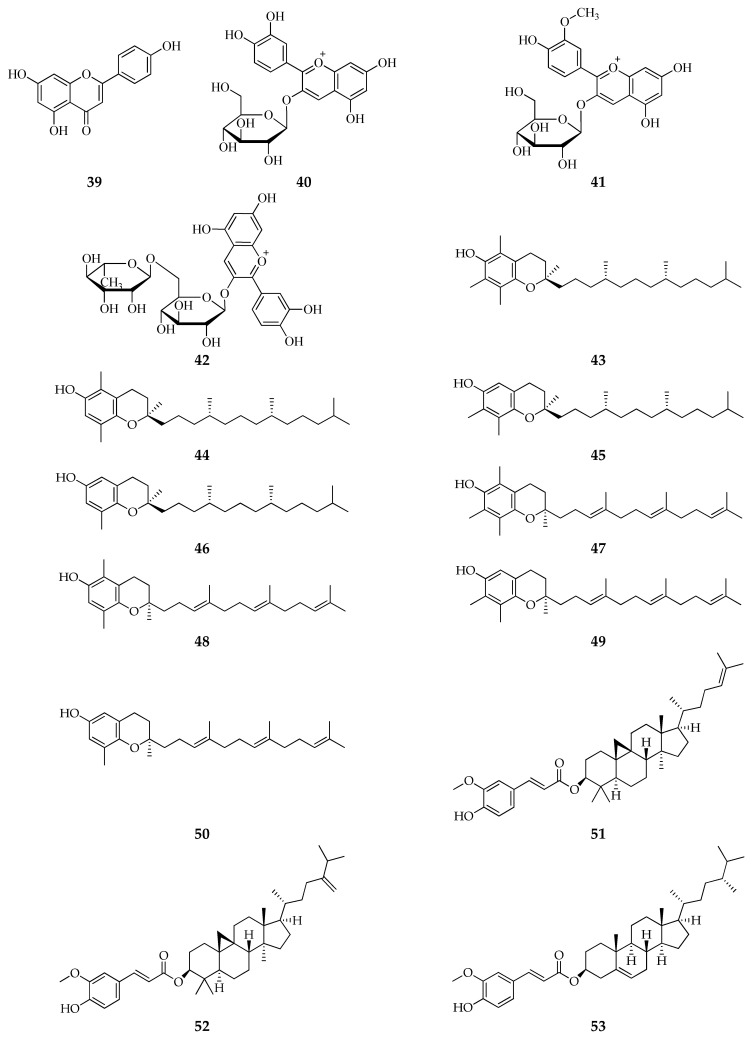

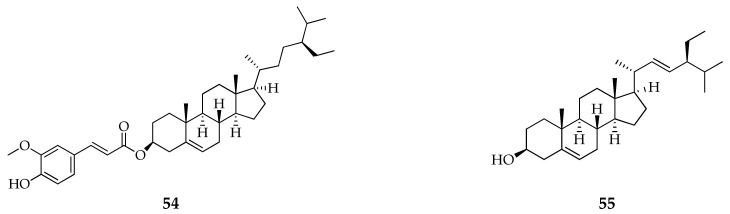
The compounds in different varieties of red rice bran use different isolation methods. (**24**) isoferulic acid; (**25**) protocatechuic acid; (**26**) *p*-hydroxybenzoic acid; (**27**) gallic acid; (**28**) vanillic acid; (**29**) syringic acid; (**30**) *p*-coumaric acid; (**31**) ferulic acid; (**32**) sinapic acid; (**33**) cinnamic acid; (**34**) protocatechualdehyde; (**35**) catechin; (**36**) quercetin; (**37**) myricetin; (**38**) luteolin; (**39**) apigenin; (**40**) cyanidin 3-glucoside; (**41**) peonidin 3-glucoside; (**42**) cyanidin 3-rutinoside; (**43**) α-tocopherols; (**44**) β-tocopherols; (**45**) γ-tocopherols; (**46**) δ-tocopherols; (**47**) α-tocotrienols; (**48**) β-tocotrienols; (**49**) γ-tocotrienols; (**50**) δ-tocotrienols; (**51**) cycloartenyl ferulate; (**52**) 24-methylene cycloartanyl ferulate; (**53**) campesteryl ferulate; (**54**) β-sitosteryl ferulate; and (**55**) stigmasterol.

From these methods, it is known that various varieties of red rice bran contain bioactive compounds such as phenolic acid (isoferulic acid [21], ferulic acid, p-hydroxybenzoic acid, vanillic acid, protocatechuic acid, syringic acid, p-coumaric acid, sinapic acid, cinnamic acid, and gallic acid) [43,44,45,46], protocatechualdehyde [44], flavonoid (catechin, quercetin, myricetin, luteolin, and apigenin) [46], anthocyanins (cyanidin-3-glucoside, peonidin 3-glucoside, and cyanidin 3-rutinoside) [44,45], vitamin E (α-tocopherols, α-tocotrienols, β-tocopherols, β-tocotrienols, γ-tocopherols, γ-tocotrienols, δ-tocopherols, and δ-tocotrienols) [44,45,47], γ-oryzanol (cycloartenyl ferulate, 24-methylenecycloartanyl ferulate, campesteryl ferulate, and β-sitosteryl ferulate) [48], and stigmasterol [35]. In contrast to white rice bran, pigmented bran such as red rice bran contains anthocyanin compounds [28,44,45] (Figure 3).

### 3.3. Black Rice Bran

Another type of pigmented rice is black rice. After the rice milling process, black rice and bran will be produced. A study used the ultrasound-assisted extraction (UAE) method to isolate compounds in black rice bran obtained from Manipur, India. The extraction process was carried out using an ultrasonic water bath equipped with a digital timer and temperature controller. Ethanol was used as a solvent. The contents of compounds in black rice bran were analyzed using GC-MS (Gas Chromatography-Mass Spectrometry). Ultimate 3000 Liquid Chromatography Systems with a UV detector was used to identify β-carotene, tocopherols, and tocotrienols. Meanwhile, phenolic acids and anthocyanins were analyzed using HPLC [49].

Black rice bran (Kamklaing) from Thailand was macerated with 1:4 (*w/v*) ethanol as solvent then filtered, and the supernatant was evaporated and lyophilized. The dry extract obtained was analyzed using HPLC to determine its phenolic acid and anthocyanin content [50]. Another extraction method to determine the anthocyanin content is conventional extraction using methanol and microwave-assisted subcritical water extraction (MA-SWE). The advantage of the MA-SWE method compared with conventional extraction using methanol is the accurate temperature control [51].

Li et al. [52] used a combination of high-speed countercurrent chromatography with reversed-phase C18 column chromatography to isolate cyanidin-3-O-β-D-glucoside. This isolation method was able to produce up to 16-fold more isolates [52]. Hou et al. [53] determined the anthocyanin content of Heiyounian 96 black rice bran obtained from Japan. The bran samples were extracted using a 1:8 (*w/v*) acidified methanol solvent for 1 h. The methanol solvent was acidified using 1.0 M HCl in a ratio of 85:15 (*v/v*). Anthocyanin content was analyzed using HPLC [53]. In addition, there are also those who use 70% methanol in 1% HCl as a solvent for extraction.

Free phenolic compounds in black rice bran can be extracted using 95% methanol acidified using 1 M HCl 1:100 (*w/v*). The ratio of methanol and HCl was 85:15 (*v/v*). Meanwhile, the bound phenolic extract was extracted from the free phenolic extract using sodium hydroxide for 1 h while stirring. Lipids were extracted using hexane. The mixture was extracted five times using ethyl acetate [54].

Black rice bran samples were determined for flavonoid content using a reversed-phase HPLC with a photodiode array detector and a combined electrospray ionization tandem mass spectrometer. Rice bran samples were prepared using 0.1% formic acid in methanol as solvent, then they were sonicated for 60 min and centrifuged. The supernatant was evaporated to a volume of 2 mL [55].

Pratiwi et al. [56] reported the content of peonidin 3-glucoside and cyanidin 3-glucoside in samples of Cempo Ireng Black rice bran from DIY, Indonesia. Rice bran samples were macerated using methanol-HCl 1:10 (*w/v*) for 48 h. The ratio of methanol and HCl was 85:15 (*v/v*). Maceration was carried out 2 times and filtered. The obtained filtrate was evaporated to obtain a dry extract. The methanol extract was redissolved with methanol and fractionated. Thin layer chromatography (TLC) method with buthanol:acetic acid:water 4:1:5 (*v/v*) as mobile phase was used for isolation. Another method used to identify the content of peonidin 3-glucoside and cyanidine 3-glucoside is Ultra High Performance Liquid Chromatography [56].

Vardhani et al. [57] carried out the extraction of black rice bran using 96% ethanol 1:4 (*w/v*) to obtain a high content of γ-oryzanol [57]. In addition, the bran also contains terpenoid compounds that give the rice a good aroma character. The analysis was carried out on glutinous and non-glutinous black rice bran samples. For sampling, the SPME device with a polydimethylsiloxane (PDMS) fiber was used. The bran sample was heated at 100 °C for 30 min. The aromatic compounds were identified using the GC-MS and GC×GC-MS methods [58].

**Figure 4:**
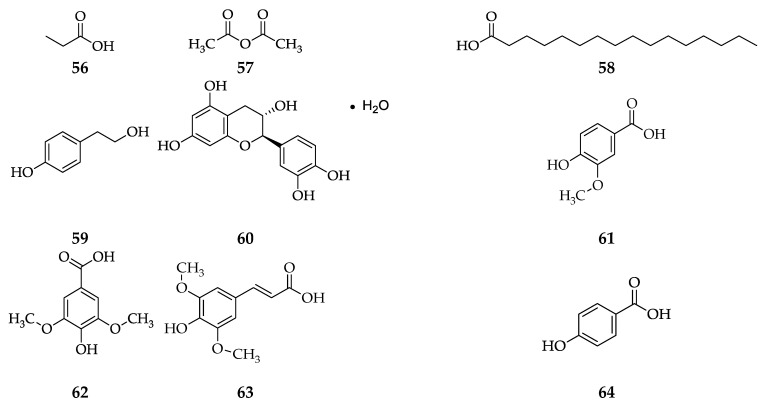

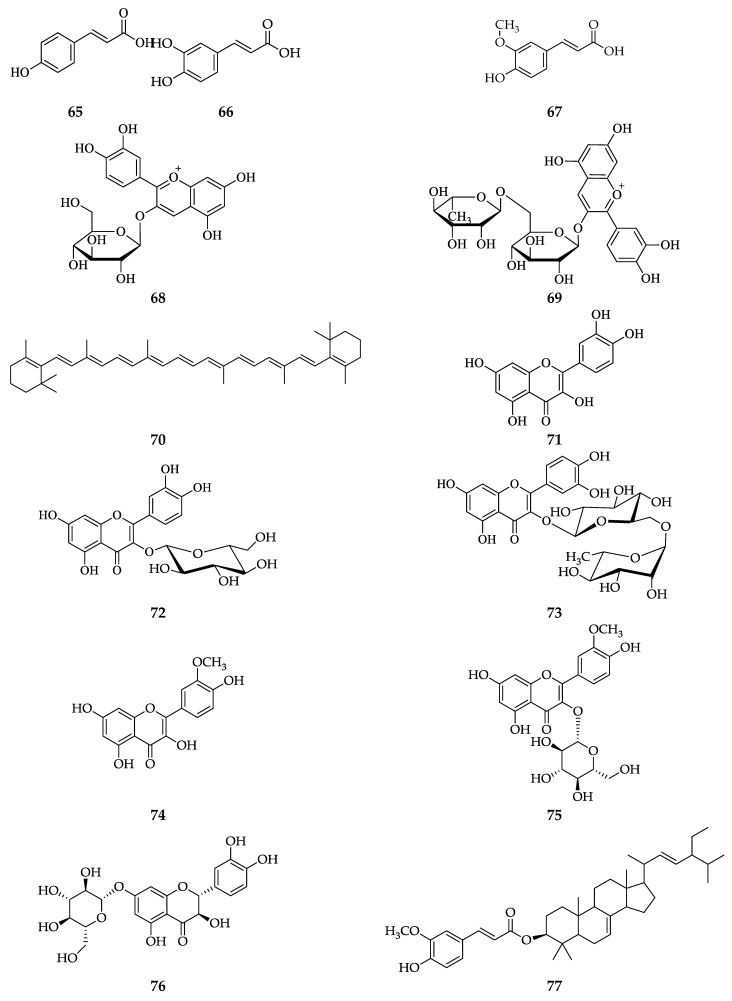

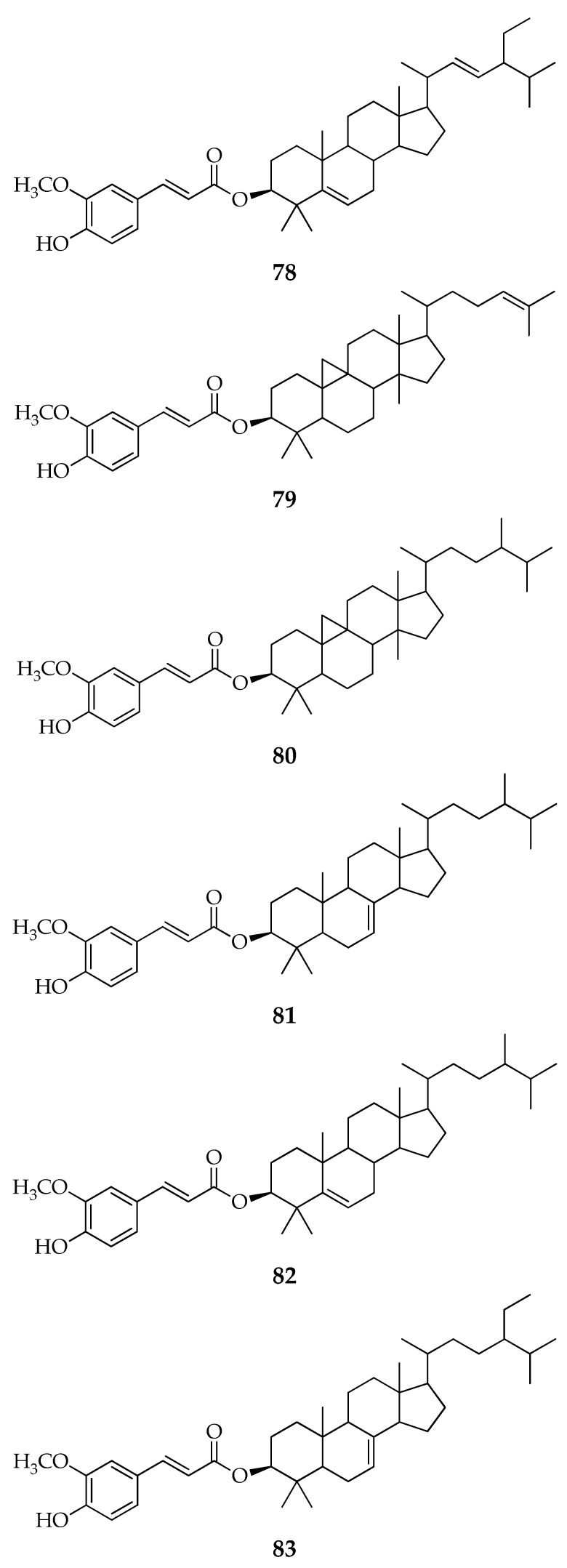

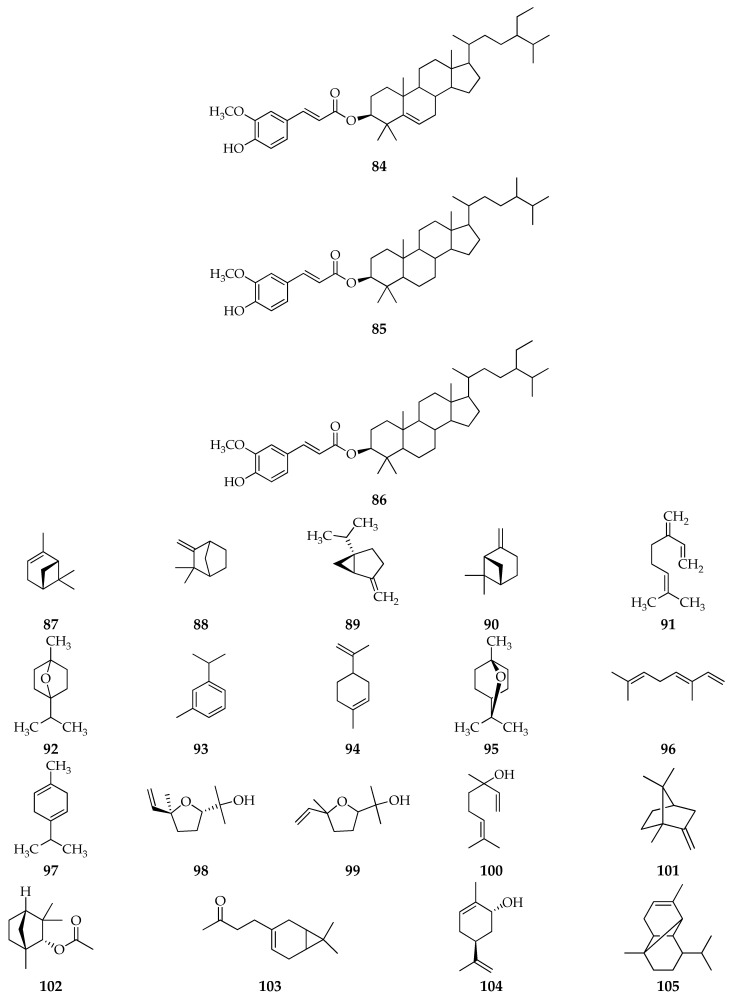

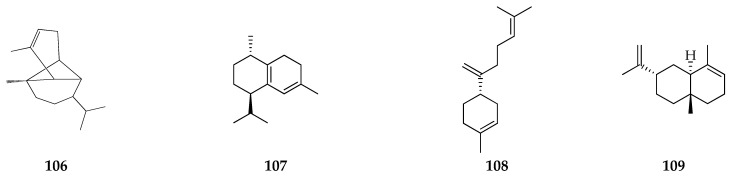
The compounds in different varieties of black rice bran use different isolation methods. (**56**) propanoic acid; (**57**) acetic anhydride; (**58**) hexadecanoic acid; (**59**) *p*-tyrosol; (**60**) catechin hydrate; (**61**) vanillic acid; (**62**) syringic acid; (**63**) sinapic acid; (**64**) 4-hydroxybenzoic acid; (**65**) *p*-coumaric acid; (**66**) caffeic acid; (**67**) ferulic acid; (**68**) cyanidin 3-glucoside; (**69**) cyanidin 3-rutinoside; (**70**) β-caroten; (**71**) quercetin; (**72**) quercetin-3-O-glucoside; (**73**) quercetin-3-O-rutinoside; (**74**) isorhamnetin; (**75**) isorhamnetin-3-O-glucoside; (**76**) taxifolin-7-O-glucoside; (**77**) Δ^7^-Stigmastenylferulate; (**78**) stigmasteryl ferulate; (**79**) cycloartenyl ferulate; (**80**) 24-methylene cycloartenol ferulate; (**81**) Δ^7^-Campestenyl ferulate; (**82**) campesteryl ferulate; (**83**) Δ^7^-Sitostenyl ferulate; (**84**) sitosteryl ferulate; (**85**) campestanyl ferulate; (**86**) sitostanyl ferulate; (**87**) α-pinene; (**88**) camphene; (**89**) sabinene; (**90**) β-pinene; (**91**) myrcene; (**92**) 1,4-cineol; (**93**) β-cymene; (**94**); limonene; (**95**) 1,8-cineol; (**96**) *trans*-β-ocimene; (**97**) γ-terpinene; (**98**) *trans*-linalool oxide; (**99**) *cis*-linalool oxide; (**100**) linalool; (**101**) camphor; (**102**) fenchyl acetate; (**103**) 10-(Acetylmethyl)-3-carene; (**104**) carveol; (**105**) α-copaene; (**106**) α-ylangene; (**107**) *trans*-cadina-1(6),4-diene; (**108**) β-bisabolene; and (**109**) 7-epi-α-selinene.

From several isolation methods and different types of black rice bran varieties, it is known that the compounds contained in black rice bran are propanoic acid, acetic anhydride, hexadecanoic acid, *p*-tyrosol, phenolic acid (catechin hydrate, vanillic acid, syringic acid, sinapic acid, 4-hydroxybenzoic acid, *p*-coumaric acid, caffeic acid, and ferulic acid), anthocyanin (cyanidin-3-rutinoside and cynanidin-3-glucoside), β-carotene, tocols (α-tocopherol and D-α-tocotrienol) [49,50,51,52,53], flavonoids (quercetin, quercetin-3-O-rutinoside, quercetin-3-O-glucoside, isorhamnetin, isorhamnetin-3-O-glucoside, and taxifolin-7-O-glucoside) [55], γ-oryzanol (Δ^7^-stigmastenyl ferulate, stigmasteryl ferulate, cycloartenyl ferulate, 24-methylene cycloartenol ferulate, Δ^7^-campestenyl ferulate, campesteryl ferulate, Δ^7^-sitostenyl ferulate, sitosteryl ferulate, campestanyl ferulate, and sitostanyl ferulate) [57], and terpenoids (α-pinene, sabinene, camphene, β-pinene, β-cymene, 1,4-cineol, myrcene, limonene, 1,8-cineol, *trans*-β-ocimene, *trans*-linalool oxide, γ-terpinene, *cis*-linalool oxide, camphor, fenchyl acetate, linalool, 10-(Acetylmethyl)-3-carene, carveol, α-copaene, α-ylangene, β-bisabolene, *trans*-cadina-1(6),4-diene, and 7-epi-α-selinene) [58] (See Figure 4).

## 4. Extraction/Isolation Methods Using Non-Toxic Solvent

Technological advances allow the extraction process to be carried out without toxic solvents. Modern techniques allow the extraction process to be faster and more efficient because it does not use a lot of solvents. The ultrasound extraction (UAE) method is an intensification technique that has been widely used in the food and pharmaceutical industry. The principle of this extraction technique is the acoustic cavitation phenomenon which causes the release of bioactive compounds [32].

Another modern extraction technology is supercritical fluid extraction (SFE). The principle of this extraction method is using pressurized liquid as solvent. Pressure, temperature, and fluid composition affect the intermediate properties between gases and liquids of supercritical fluids. In particular, the viscosity and surface tension are similar to those of liquids. At the same time, the diffusion coefficient is similar to that of a gas, which allows for more efficient extraction by dispersing more rapidly through the solid matrix [32].

## 5. Rice Bran Bioactivity and Test Methods

### 5.1. Antimicrobial

Rice bran is known have antimicrobial activity. This antimicrobial activity is used in the food and pharmaceutical fields. A study reported that the ethanolic extract of rice bran had antimicrobial activity on emulsion-type mayonnaise. In this study, antibacterial analysis was performed using the broth microdilution method with Listeria innocua (CECT 910) and Escherichia coli (CECT 45) bacteria as strains tested [22]. Taniguchi et al. [59] carried out the synthesis of multifunctional cationic peptides identified in rice bran. The peptides were LRRHASEGGHGPHW, EKLLGKQDKGVIIRA, and SSFSKGVQRAAF. The results showed that the three peptides had antimicrobial, lipopolysaccharide-neutralizing, and angiogenic activities. The pathogens used in the antimicrobial activity test were a Gram-negative bacterium (Porphyromonas ginigivalis ATCC 33277), Gram-positive bacteria (Propionibacterium acnes JCM 6473 and Streptocossus mutans JCM5705), and a fungus (Candida albicans NBRC 1385). Antimicrobial activity assays were performed using 96-well plates [59].

Polysaccharides in rice bran are also known to have antimicrobial activity [60]. Polysaccharides extracted using hexane have better antimicrobial activity than those extracted using ethanol. The bacteria used for antimicrobial testing were Staphylococcus aureus (TISTR 517), Bacillus cereus (TISTR 687), and Escherichia coli (TISTR 1261) strains [60]. In addition to the polysaccharide content which has antimicrobial activity, the content of phenolic compounds in bran also has antimicrobial activity. Achmad et al. [61] used the bacterium Porphyromonas gingivalis to test the antimicrobial activity of rice bran using the disc diffusion method [61]. The content of γ-oryzanol in rice bran also has antimicrobial activity. The bran was extracted using isopropanol 1:25 (*w/v*), then the extract was dissolved using a 30% ethanol solution. This extract solution was then added to the Tryptic soy broth (TSB) medium. Antibacterial activity was carried out using the microdilution method on Gram-negative bacteria: resistant Escherichia coli, Escherichia coli (ATCC 25922), Pseudomonas aeruginosa (ATCC 27853), and two Gram-positive bacteria: MRSA (Methicillin-resistant Staphylococcus aureus) and Staphylococcus aureus (ATCC 11632). In addition, an antifungal activity was tested using the microdilution method on Aspergillus fumigatus (ATCC 9197), Penicillium ochrochloron (ATCC 9112), Aspergillus niger (ATCC 6275), and Penicillium funiculosum (ATCC 36839) [62].

Phenolic extract from rice bran that fermented with Rhizopus oryzae was used to increase the shelf life of pizza dough. The results showed the phenolic extract that was able to increase the shelf life of pizza dough with good antifungal activity as glucosamine 17.66 µg g*^−^*^1^, score of molds and yeasts 1.3 × 10^1^ CFU g*^−^*^1^, and invertase enzyme activity in 0.04 mg min. proteins*^−^*^1^ [63]. Moon et al. [64] reported that rice bran fermented using Lactiplantabillus plantarum EM had high antimicrobial activity. The antimicrobial activity test method used was spot-on-the-lawn assays. This research uses foodborne pathogenic bacteria and food spoilage fungi. The results showed high antimicrobial activity of 100–400 AU/mL [64].

Rice bran is also known to inhibit Salmonella colonization in mice. This study could be useful for protection against enteric pathogens. The results showed that rats fed a diet for one week containing 10 and 20% rice bran showed a decrease in the excretion of Salmonella feces [65]. In addition, bioprocessed (fermented) rice bran extract (BPRBE) from liquid mycelia culture of Lentinus edodes was used to inhibit Salmonella Typhimurium (SL1344). The mechanism of resistance against bacteria is by promoting upregulation of protein expression that induces bacterial destruction in autolysosomes of RAW 264.7 cells [66].

Fermented bran is known to have phenolic compounds that can inhibit fungal growth. Tests for antifungal activity were carried out in Petri dishes. The media consisted of potato dextrose agar (PDA), Penicillium verrucosum, (4 × 10^6^ spores mL*^−^*^1^), and the preservatives (167 g mL*^−^*^1^). The results showed that the inhibition of fungi was 39.8% [67]. Rice bran fermented using Rhizopus oryzae CECT 7560 contains phenolic compounds that can inhibit fungal growth. A study reported that Fusarium, Aspergillus, and Penicillium strains were able to be inhibited by phenolic extracts from fermented rice bran. In addition, this antifungal activity was applied to bread contaminated with P. expansum. The resulting reductions were 0.6 and 0.7 log CFU g*^−^*^1^ [68].

### 5.2. Antioxidant

Research on antioxidant activity in rice bran has been widely reported. Rice bran extract using 80% methanol has the highest antioxidant activity compared with using other solvents such as 100% methanol, 80% acetone, and 100% acetone. The antioxidant activity testing methods were carried out in different systems, namely in a linoleic acid system, in a β-carotene linoleic acid system, and using sunflower oil as oxidation substrate [69].

In general, many antioxidant activity analysis methods use the DPPH radical (2,2-diphenyl-1-picrylhydrazyl) because it is stable. The principle of this method is that the DPPH color changes from purple to yellow when it receives a hydrogen atom donor from a bioactive compound. In addition, another method that is widely used for the analysis of antioxidant activity is the ABTS (2,2’-azino-bis (3-ethylbenzothiazoline-6-sulfonic acid) method. The principle of this method is to reduce the intensity the blue color of the ABTS radicals in the presence of bioactive antioxidant compounds [70].

Another study tested the antioxidant activity of rice bran using different methods such as DPPH radical scavenging ability [21,71,72,73,74,75,76,77,78,79], ABTS radical scavenging assays [45,71,79], Total antioxidant activity (TAA) [72], Nitic oxide (NO) scavenging ability [45,72], ferric reducing antioxidant power (FRAP) assay [45,74], Hydroxyl Radical Scavenging Activity [77], Superoxide Anion-Scavenging Activity [45,77], Oxygen Radical Absorbance Capacity (ORAC) assay [21], and inhibition of lipid peroxidation [45].

Several studies have shown that there is a relationship between total phenolic content and antioxidant activity. The higher the total phenolic content, the higher the antioxidant activity [45,54,80].

### 5.3. Cancer Chemopreventive

The process of preventing cancer using chemicals is called cancer chemoprevention [81]. Prevention, inhibition, and decrease of cancer growth can use natural products such as rice bran. Several studies have been conducted in vivo and in vitro on cancer chemopreventive activity in rice bran [82]. Colorectal cancer cells (CRC) can be inhibited with red, brown, and purple rice bran from several varieties. The results showed the influence of total phenolic content and γ-tocotrienol on the inhibition of colorectal cancer cell growth (CRC) [83]. In addition, the content of cycloartenyl ferulate also has a role in inhibiting the growth of human colorectal adenocarcinoma SW480 [84]. Peptides purified from rice bran are also known to have activity as an inhibitor of cancer growth in colon, lung, liver, and breast cancer cells [85].

Red rice bran is known to have strong activity in inhibiting cervical, stomach, and leukemia cancer cells. The test was carried out using the MTT assay. The MTT (3-[4,5-dimethylthiazol-2-yl]-2,5 diphenyl tetrazolium bromide) assay is based on the conversion of MTT into formazan crystals by living cells, which determines mitochondrial activity. The content of proanthocyanidins in red rice bran has a role in inhibiting the growth of cancer cells [86]. Another study showed that purified and fractionated polysaccharides from rice bran had antitumor activity. This study used RAW 264.7 macrophages and melanoma cell line B16. The mechanism of polysaccharides in antitumor activity is by enhancing immune function [87]. The ethanol extract fraction of the black rice bran of Woja Laka variety also had chemopreventive activity on liver carcinoma HepG2 cells which was carried out using the MTT assay. Its mechanism of action is by inhibiting cell proliferation, inducing apoptosis, and causing G0/G1 phase termination in HepG2 cells [88].

In vivo studies showed that phytic acid extract from rice bran was able to suppress colon carcinogenesis in rats. The concentration of 0.2% (*w/v*) phytic acid extract given to rats that had been induced by azoxymethane (AOM) was able to reduce the formation of ACF [89].

### 5.4. Antidiabetic

Another bioactivity that rice bran has is antidiabetic. Antidiabetic test was carried out on rice bran extracted with food grade glycerol. Antidiabetic testing was carried out using the digestive enzymes inhibition assays method and also looked at AGEs (Advanced Glycation End Products) formation and inhibition. The results showed that rice bran extract was able to inhibit the activity of α-amylase, α-glucosidase, and lipase enzymes by 34.2%, 29.9%, and 12.4%, respectively. In addition, the bran extract was able to inhibit the formation of AGEs by 52.6% at a concentration of 20 L/mL [90].

The content of momilactone A (MA) and momilactone B (MB) in rice bran of the Koshihikari variety has a role in inhibiting pancreatic α-Amylase and α-Glucosidase. α-Amylase and α-Glucosidase inhibition assays were performed using a Microplate Spectrophotometer. Momilactone A (MA) and momilactone B (MB) samples were incubated with the enzyme and substrate in each well of a microplate, then the percent inhibition was calculated. The results showed the IC_50_ values of a-amylase inhibition (mg/mL) using momilactone A and B were 132.56 and 129.02, respectively. Meanwhile, the IC_50_ values of a-glucosidase inhibition (mg/mL) using momilactone A and B were 991.95 and 612.03, respectively [91]. Another study made gluten-free composite flours from wheat (Triticum aestivum), soy (Glycine max), oat bran (Avena sativa), and rice bran (Oryza sativa). The gluten-free composite flour extract was tested for its activity in inhibiting α-amylase and α-glucosidase enzymes. Then, in vivo testing was carried out on the glycemic index and glycemic load of the formulated composite flour samples. The results showed that the inhibition of α-amylase and α-glucosidase enzymes was 66.75% and 57.40%, respectively, while the results of the glycemic load (GL) of formulated foods and control samples ranged from 24.64 to 28.5%. The glycemic index of foods in the present study varied from 40.03 to 46.41%. The activity of lowering blood glucose levels showed high results of 79.62% [92].

Egyptian rice bran was stabilized and extracted, with extract standardized to contain 2% γ-oryzanol. In addition, the rice bran extract contains γ-tocotrienol, policosanol, and γ-oryzanol. The extract was tested for insulin secretion in vitro using INS-1 cells. The results showed that rice bran extract was able to increase insulin secretion. The higher the concentration of the bran extract used, the higher the insulin secretion. The results also showed that insulin secretion was induced by the content of policosanol and γ-oryzanol. In vivo experiments GTT (Glucose Tolerance Test) in rats were then performed; the results showed that plasma insulin increased after administration of 10 mg/kg rice bran extract given orally [93]. The polyphenol content in rice bran also has therapeutic potential against type 2 diabetes mellitus [94].

Stabilized rice bran (SRB), rice bran water solubles (RBWS), and rice bran fiber concentrates (RBFC) were administered to patients with insulin-dependent and noninsulin-dependent diabetes mellitus (Type I and Type II) for 60 days. The results showed a decrease in the fasting serum glucose levels and an increase in serum insulin levels. Rice bran water solubles (RBWS) showed the best results in decreasing the fasting serum glucose levels and increasing serum insulin levels [95]. Patients with type 2 diabetes mellitus who were given a supplement of 20 g stabilized rice bran once a day for 12 weeks showed lower postprandial glucose than patients with type 2 diabetes mellitus who were given only a placebo [96]. Dietary supplementation with oil-soluble rice bran triterpenoids in healthy male adults was able to reduce blood glucose increases in subjects with higher postprandial blood glucose elevations. Self-monitoring devices were used to determine blood glucose levels. The blood samples measured were fasting blood samples and 240 min after eating [97]. Rice bran milk given to primary school teachers in the city of Makassar was able to reduce fasting blood glucose levels and body weight. Rice bran milk is given as much as 30 g once a day for 1 month [98]. Triterpene alcohol and sterol preparation (TASP) from rice bran was able to reduce blood glucose levels in mice and humans [99].

### 5.5. Hypolipidemic Agents

Rice bran has also been studied to have bioactivity as a hypolipidemic. The tocotrienol-rich fraction isolated from rice bran oil was able to reduce lipid levels in rats. Mice with induced hyperlipidemia from being given tocotrienol-rich fraction (TRF) supplements for three weeks were able to reduce lipid parameters such as plasma triglycerides, plasma cholesterol, LDL cholesterol, HMG-CoA reductase, TBARS, and conjugated dienes. The optimal effect of TRF administration is at a dose of 8 mg TRF/kg/day [100]. Kang et al. [101] used mice that had been fed a high-fat diet resulting in an increase in lipid parameters. The mice were then fed with the addition of rice bran and phytic acid for 7 weeks. The addition of rice bran and phytic acid to food was able to reduce triglyceride and total cholesterol levels in plasma and liver which were determined using a commercial kit (Asan Pharmaceutical, Seoul, Korea). Meanwhile, the content of triglycerides and total cholesterol in the feces increased. Rice bran and phytic acid decreased fat content by increasing fecal lipid excretion [101].

The aqueous enzymatic extract from rice bran (AEERB) has hypolipidemic activity and enhances antioxidant status. Hypolipidemic testing was carried out by in vivo on male wistar rats. Rice bran was hydrolyzed in a water bath using the enzyme trypsin at a temperature of 56 °C and pH 7,9. AEERB contains protein, γ-oryzanol, and tocol. Automatic chemistry analyzer was used to analyze metabolic parameters such as the concentrations of TC, triglyceride (TG), LDL-C, HDL-C, ApoA, ApoB, and Lp (a) in the serum lipids. Furthermore, ELISA (enzyme-linked immunosorbent assay) methods with commercial kits were used to determine the hepatic HMG-CoA reductase activity. The results showed decreased liver lipid levels, inhibition of hepatic 3-hydroxyl-3-methylglutaryl CoA reductase activity, and increased fecal excretion of total lipid and total cholesterol (TC) (*p* < 0.05) after administration of AEERB to male wistar rats that had been treated and fed a diet high in fat and cholesterol [102].

Feeding rice bran phenolic extracts (RBPE) for 6 weeks (200 and 400 mg/kg/d) to high-fat (HF)-diet -induced mice was able to reduce the increase in levels of TC, TG, LDL-c, and FFA and reduced the decrease in HDL-c in serum. In addition, RBPE feeding was able to increase adiponectin levels in serum which decreased after high fat (HF) diet was induced. The results of histological analysis of liver tissue using hematoxylin and eosin staining and Oil Red O staining showed a reduction in lipid droplets after RBPE feeding. In addition, RBPE is also able to inhibit SREBP1c nuclear protein translocation and promote PPARα activation. Quantification of gene expression in the liver with RT-qPCR showed that RBPE was able to inhibit the expression of the HMGR gene in which the HMGR enzyme catalyzes the reduction of HMG-CoA to mevalonic acid. In addition, RBPE also inhibited the expression of SREBP1c, SCD, and ACC activation but increased the expression of CPT1a, CYP7A1, and AMPKα proteins [103].

Justo et al. [104] used obese Zucker rats that were given rice bran enzymatic extract (RBEE) 1 and 5%. The results showed a reduction in circulating levels of TG and TC and an increase in HDL-C. RBEE was also able to reduce hypertension in obese Zucker rats [104]. The rice bran enzymatic extract (RBEE) which contains γ-oryzanol, phytosterols, and tocotrienols has hypolipidemic activity by increasing HDL-C levels in serum and increasing total cholesterol and AST. RBEE was able to inhibit HMG-CoA reductase activity and increase cholesterol excretion. In addition, RBEE was also able to reduce lipid deposition and macrophage infiltration in the aortic sinus. The expression of ICAM-1 and VCAM-1 was also able to be inhibited by RBEE. In this study, we used RBEE with a dose of 1 and 5%. According to this study, regular RBEE supplementation was able to reduce the development of plaque and hepatic steatosis [105,106]. A rice bran oil (RBO) diet in type-2 diabetic rats induced by streptozotocin/nicotinamide had lower plasma triglyceride levels than in rats fed a control diet. The content of γ-oryzanol and γ-tocotrienol in rice bran oil was able to suppress hyperlipidemia in type-2 diabetic rats [107].

Clinical studies were also conducted on overweight and obese adults who were not taking cholesterol-lowering drugs. Participants for 8 weeks were given a diet containing either pigmented rice bran (RB) or the RB with addition of plant sterols (RB + PS) snack bars. The results showed a weight loss of about 4.7 ± 2.2 kg. Total cholesterol content decreased by 36 ± 25 g/dL (for RB + PS) and 7 ± 16 g/dL (for RB). Meanwhile, LDL content decreased by 22.3 ± 25.2 g/dL (for RB + PS) and 4.4 ± 18.9 g/dL (for RB) [108]. In addition, rice bran extract containing the acylated steryl glucoside fraction was able to reduce LDL-c levels in the blood when was tested on obese men in Japan [109].

## 6. Phenolic Compound in Rice Bran as Antioxidant Properties

A group of small molecules having at least one phenol unit are phenolic compounds. Phenolic compounds are divided into different subgroups such as phenolic acids, flavonoids, tannins, coumarins, lignans, quinones, stilbenes, and curcuminoids based on their chemical structure [110]. Phenolic compounds are known to have very good antioxidant activity [111]. The presence of a hydroxyl group on the aromatic ring of phenolic compounds allows phenolic compounds to act as antioxidants. The hydroxyl group is able to transfer hydrogen atoms to a free radical so that it is stabilized [36]. Rice bran has the highest total phenolic content (TPC) compared with whole grain, embryo, and endosperm in white, red, and black rice [21].

Phenolic compounds in rice bran exist in bound, conjugated, and free forms [112]. Free phenolics are free soluble compounds that are localized in plant cell vacuoles where they are trapped. Meanwhile, conjugated phenolics are soluble phenolics that are covalently bonded to other molecules such as fatty acids [113]. Conjugated phenolics are soluble components that can be extracted by aqueous solutions of methanol but are considered to be bound to soluble oligosaccharides and peptides through ester bonds or ether bonds, which can be released after hydrolysis [114]. Bound phenolics are insoluble components because they are bound to cell wall components such as pectin, cellulose, arabionoxylan, and structural proteins by covalent bonds. The bound phenolic content is usually higher than the free soluble phenolic [113,115]. Studies have been conducted which showed that bound and insoluble phenolic extracts in corn, wheat, oats, and rice have higher antioxidant activity than soluble free phenolic extracts [115]. Unfortunately, insoluble bound phenolic compounds are more difficult to extract; in the extraction process, hydrolysis is required first, such as by using alkaline and enzymes [26,116,117]. Therefore, currently there are many studies that focus on increasing antioxidants in rice bran with various methods.

## 7. Enhancement of The Antioxidant Capacity Method in Rice Bran

In plants, the contents of bioactive compound exist in free and bound forms. Previous studies reported that fermentation and treatment with enzymes can increase the contents of bioactive compounds in rice bran. Fermentation process and treatment with enzymes are capable of releasing bioactive compounds that bound with cell wall components, such as lignins [27,116]. The methods used to increase antioxidant capacity in rice bran from several rice varieties are summarized in Table 1.

## 8. Conclusions

Bioactive compounds in white, red, and black rice bran can be isolated by column chromatography using silica gel. The solvents used for the extraction process are methanol, ethanol, acetone, chloroform, hexane, ethyl acetate, dichloromethane, and isopropanol. However, these solvents are toxic so that in the future alternative extraction methods such as ultrasound extraction (UAE) and supercritical fluid extraction (SFE) can be used. Several methods of identification and characterization of bioactive compounds in rice bran used HPLC (High Performance Liquid Chromatography), TLC (Thin Layer Chromatography), FTIR (Fourier-transform infrared spectroscopy), and NMR (Nuclear magnetic resonance). The process of releasing bonds between the components of bioactive compounds and cell wall components affects the amount of bioactive compounds extracted so that the antioxidant activity of rice bran increases. Fermentation method with *Lactobacillus plantarum, Lactobacillus lactic, Lactobacillus casei, Rizhopus oryzae, Bacillus subtilis, Aspergillus oryzae, Rhizopus oligosporus* (strain F0020), *Monascus purpureus* (strain F0061), and *Saccharomyces cerevisiae* have been used on rice bran. Treatment using enzymes carbohydrases, cellulase, xylanase, trypsin, chymotrypsin, papain, bromelain, or flavorzyme, fiberzyme, and endo-1,4-beta-xylanase has been carried out. In the future, methods such as infrared radiation heating, which is easier and can be applied on an industrial scale, can be developed to increase the antioxidant capacity of rice bran.

## 9. Patents

This section is not mandatory but may be added if there are patents resulting from the work reported in this manuscript.

## Figures and Tables

**Table 1:** Enhancement of The Antioxidant Capacity Method in Rice Bran.

Variety (Source)	Method	Effect	Reference
*Japonica* type variety unknown (Seoul, Korea)	treated with different carbohydrases (Viscozyme, Termamyl, Celluclast, AMG, Ultraflo, and Pentopan)	ethanol extract (%), reducing sugar (mg/g), and total phenolic content (mg GAE/g) increased	[118]
*Jyothi* variety red rice paddy (Karnataka, India)	treated with *Bacillus* Endoxylanase (EXB) and *Trichoderma* Endoxylanase (EXF)	total flavonoid; individual soluble phenolic components cycloartenyl ferulate; β-sitosteryl ferulate; and δ, γ, α-tocotrienols and tocopherols increased	[48]
Unknown (Yogyakarta, Indonesia)	fermented with *Lactobacillus plantarum* and *Lactobacillus lactic*	total phenolic content (mg GAE/g dw) increased	[119]
Khao Bahn Nah and Thai jasmine (Prachin Buri province, Thailand)	fermented with *Lactobacillus casei* and *Lactobacillus plantarum*	polysaccharide (mg/mL) decreased and total phenolic content (mg/mL) increased	[120]
BR-IRGA 417 (Brazil)	fermented with *Rizhopus oryzae*	total phenolic content (mg/g) increased	[121]
Unknown (Stuttgart, Ark., U.S.A.)	fermented with *Bacillus subtilis* subspecies *subtilis* NRRL NRS-744	total phenolic content (mg FAE/g) increased	[122]
PB-1121 (Sirsa, India)	solid-state fermentation (SSF) with *Aspergillus oryzae* MTCC 3107	total phenolic content (g GAE/g dwb) and condensed tannin content (mg CE/g dwb) increased	[123]
*IR 64* and *Jyothi* (Karnataka, India)	treated with Cellulase from *Aspergillus niger* (1.4 U/mg solid) from Sigma-Aldrich, USA and Xylanase D-762-p (400 U/g solid) from Biocatalyst	γ-oryzanol; soluble, bound, and total polyphenols; flavonoid and tannin content increased	[124]
RD6 (Chiang Mai Rice Research Center, Thailand)	Three different methods were used for comparison, namely enzymatically stabilized rice bran (ESRB) using (trypsin, chymotrypsin, papain, bromelain, or Flavorzyme) enzymes; raw rice bran (RRB) without any treatment; and thermally stabilized rice bran (TSRB) which is raw rice bran heated at 100 °C in open steam for 15 min	free phenolics, γ-oryzanol, tocopherols, and tocotrienols content in ESRB is higher compared with TSRB and RRB	[125]
*Jyothi* paddy (Karnataka, India)	Rice bran was treated with a combination of endo-1,4-beta-xylanase (EXYL) and Fiberzyme (Fzyme) enzymes. Three combinations of the two enzymes were performed, namely 1.5 BGU + 3 EXU (CXC1), 3 BGU + 2 EXU (CXC2), and 4.5 BGU + 1 EXU (CXC3) enzymes.	The content of ferulic acid in soluble phenolics, *p*-coumaric acid in bound phenolics, and γ-oryzanol fractions increased the most in the combination CXC2. In addition, ferric reducing power, DPPH• scavenging capacity, nitric oxide scavenging, and inhibition of human LDL oxidation were increased for all three types of enzyme combination. Superoxide anion and hydroxyl radical scavenging activities increased for the combination of CXC1 and CXC2 enzymes.	[126]
Khao Dok Mali 105 (Suphan Buri Province, Thailand)	Six different pretreatment methods were carried out in rice bran to extract rice bran oil, such as microwave heating (60–110 °C for 3 min), hot air heating (70–180 °C for 10 min), roasting (60 and 80 °C for 3 min), parboiling (75 °C for 60 min), autoclave heating (121 °C for 15 min), and hydrolysis with α-amylase 1375 units/mL under the optimum condition for enzyme activity (180 rpm, at 50 °C for 120 min)	Pretreatment with roasting at 60 °C produced the highest γ-oryzanol content which was 46.9 mg/mL of rice bran oil.	[127]
Unknown (Kuala Lumpur, Malaysia)	fermented with *Rhizopus oligosporus* (strain F0020) and *Monascus purpureus* (strain F0061)	The phenolic acid content in methanol extracts of fermented rice bran such as ferulic, sinapic, vanillic, caffeic, syringic, and 4-hydroxybenzoic acids increased	[128]
KDML 105 (Northeastern Thailand)	Three different types of pretreatment were carried out on rice bran compared with raw bran, namely hot-air (120°C for 30 min), far-infrared radiation (FIR), and hydrolysis with cellulase	There was an increase in DPPH radical scavenging activities, ferric reducing antioxidant power (FRAP), total phenolic content (TPC), several phenolic acids, α-tocopherols, γ-tocopherols, and δ-tocopherols; the highest was in rice bran treated with FIR-treated	[129]
Axios-Long A type (Unknown)	infrared radiation heating	There was an increase in phenolic content and antioxidant activity in the bound extract of rice bran which was directly proportional to the increase in IR power.	[130]
Unknown (Rio Grande do Sul, Brazil)	fermented rice bran by *Saccharomyces cerevisiae* for gluten-free cookies formulation with different fermented rice bran composition, namely 7.08%, 14.16%, and 28.33%	Cookie formulations with a fermented rice bran composition of 7.08% showed the highest phenolic compound content compared with the others and was higher than cookies without fermented rice bran.	[131]

## Data Availability

The study did not report any data.
